# Stellenwert der CT-Kolonographie bei kolorektalen Karzinomen: Eine kritische Betrachtung

**DOI:** 10.1007/s00117-025-01456-8

**Published:** 2025-05-20

**Authors:** Thomas Mang, Nino Bogveradze, Michael Bergman, Simon Leitner, Martina Scharitzer

**Affiliations:** 1 Universitätsklinik für Radiologie und Nuklearmedizin, Medizinische Universität Wien, Währinger Gürtel 18–20, 1090 Wien, Österreich; 2 Universitätsklinik für Allgemeinchirurgie, Medizinische Universität Wien, Währinger Gürtel 18–20, 1090 Wien, Österreich

**Keywords:** CT Kolonografie, Kolorektale Neoplasie, Kolorektales Adenom, Vorsorge, Nachsorge, Detektionsrate, Screening, CT colonography, Colorectal neoplasia, Colorectal adenoma, Surveillance, Follow-up, Detection rate, Screening

## Abstract

**Hintergrund:**

Die Computertomographie-Kolonographie (CTK) ist eine minimal-invasive radiologische Untersuchung zur Darstellung des gesamten Kolons und Rektums. Sie ersetzt den mittlerweile obsoleten Kolonkontrasteinlauf und stellt eine wichtige Ergänzung zur Koloskopie in der Dickdarmkrebsdiagnostik dar.

**Ziel der Arbeit:**

Ziel dieser Arbeit ist es, einen Überblick über den aktuellen Stellenwert und den Nutzen der CTK zur minimal-invasiven bildgebenden Diagnostik kolorektaler Karzinome (KRK) zu bieten.

**Material und Methoden:**

Mittels einer ausführlichen Literaturrecherche wird der aktuelle Wissensstand zur Rolle der CTK in der Dickdarmkrebsdiagnostik zusammengefasst. Dies umfasst neben der Bildgebung manifester Karzinome auch den Einsatz der CTK in der Vor- und Nachsorge des KRK.

**Ergebnisse:**

Die CTK ist eine ausgereifte, minimal-invasive Untersuchung zur Abklärung kolorektaler Neoplasien. Die Detektionsrate für gutartige Krebsvorstufen und manifeste Karzinome ist vergleichbar mit der Detektionsrate der optischen Koloskopie. Fehldiagnosen bei KRK sind selten und meist durch Perzeptionsfehler und technische Fehler bedingt. Durch die Möglichkeit bei stenosierenden Karzinomen auch endoskopisch nicht einsehbare Darmabschnitte und extrakolische Strukturen darzustellen, können zusätzlich zur Tumordetektion auch synchrone Dickdarmneoplasien erkannt und die abdominale Tumorausbreitung beurteilt werden. Neben der Diagnose manifester Karzinome kann die CTK durch das rechtzeitige Erkennen benigner Krebsvorstufen zur Dickdarmkrebsvorsorge eingesetzt werden. In der KRK-Nachsorge kann die CTK bei nicht erfolgreicher Koloskopie alternativ zur Rezidivdiagnostik eingesetzt werden.

**Diskussion:**

Die CT-Kolonographie ist die radiologische Untersuchung der Wahl zur Detektion kolorektaler Neoplasien. Sie kommt meistens bei Personen zum Einsatz, bei denen eine Koloskopie nicht möglich oder nur unvollständig ist, kann aber auch zur opportunistischen Dickdarmkrebsvorsorge eingesetzt werden.

## Abstract (en)

**Background:**

Computed tomography colonography (CTC) is a minimally invasive examination for radiologic imaging of the entire colon and rectum. It has replaced the now obsolete barium enemas and represents an important option to colonoscopy for the detection of colorectal neoplasia.

**Objectives:**

The aim of this article is to review the current status of CTC for detection of colorectal cancer (CRC) and its precursors.

**Materials and methods:**

Based on an extensive literature research, the current role of CTC in colorectal cancer imaging is summarized. This includes not only CRC detection but also the role of CTC in CRC screening and surveillance.

**Results:**

CTC is a well-developed, minimally invasive examination for the identification of colorectal neoplasia. The diagnostic accuracy for colorectal adenomas and cancer is comparable to that of optical colonoscopy. Missed CRC cases are rare and typically result from perceptual or technical errors. In addition to the detection and characterization of carcinomas, CTC enables the visualization of prestenotic colonic segments and extracolonic structures. This allows for the preoperative detection of synchronous colonic neoplasms that are not endoscopically assessable as well as evaluation of abdominal tumor spread. Due to its ability to detect benign precancerous adenomas with high sensitivity, CTC can be used for opportunistic colorectal cancer screening. In CRC follow-up, CTC may be used to diagnose recurrence if colonoscopy is incomplete or not feasible.

**Conclusion:**

CTC is the radiological examination of choice for the detection of colorectal neoplasia. It is typically used in patients where colonoscopy is incomplete, contraindicated or refused. Moreover, CTC serves as a noninvasive option for opportunistic colorectal cancer screening.

Die Computertomographie-Kolonographie (CTK), auch virtuelle Koloskopie genannt, ist ein radiologisches Verfahren zur bildgebenden Diagnostik des gesamten Dickdarms. Die Untersuchung ist minimal-invasiv und erlaubt neben der endoluminalen Darstellung des Kolons und Rektums auch die Untersuchung der perikolischen Strukturen und der abdominalen Organe. Die wesentlichen Zielläsionen der CTK sind kolorektale Karzinome (KRK) und fortgeschrittene Adenome, die zu fortgeschrittenen Neoplasien zusammengefasst werden. Die Untersuchung ist für PatientInnen sicher und wenig belastend, Komplikationen sind sehr selten.

Über 30 Jahre nach ihrer Erstvorstellung stellt die CTK heute eine methodisch ausgereifte, nichtinvasive Dickdarmuntersuchung dar. Indikationen, Untersuchungstechnik sowie Befundung sind international standardisiert und in internationalen Leitlinien veröffentlicht [1–4]. Sie wurden an anderer Stelle bereits ausführlich dargestellt [5].

Die diagnostische Performance der CTK wurde im Rahmen zahlreicher Studien sowohl bei symptomatischen PatientInnen als auch in der Vorsorge validiert. Die in einer Metaanalyse zusammengefassten Studienergebnisse haben gezeigt, dass die Erkennungsrate der CTK für kolorektale Karzinome mit 96 % der Detektionsrate der Koloskopie (95 %) ebenbürtig ist [6]. Auch in prospektiven, randomisierten Studien war die diagnostische Ausbeute beider Methoden gleich hoch [7, 8]. Das zeigt sich auch in einer vergleichbar geringen Rate an Intervallkarzinomen [9]. Die CTK vereint somit die Vorteile der minimalen Invasivität mit einer hohen diagnostischen Genauigkeit. Sie ersetzt den für diese Indikationen obsoleten Kolonkontrasteinlauf (Irrigoskopie) und ergänzt die Koloskopie [1].

In dieser Übersichtsarbeit wird die Rolle der CTK in den wesentlichen Bereichen der Dickdarmkrebsdiagnostik zusammengefasst. Neben der Diagnostik manifester Karzinome werden auch relevante Aspekte zum Einsatz in der Vor- und Nachsorge des KRK diskutiert.

## Hintergrund

Das kolorektale Karzinom ist in Europa bei Frauen das am zweit- und bei Männern das am dritthäufigsten auftretende Karzinom [10]. Unter den Krebserkrankungen hat es die zweithöchste Mortalität bei Männern und die dritthäufigste bei Frauen.

Kolorektale Karzinome werden am häufigsten im Rahmen einer Koloskopie diagnostiziert, die entweder zur Abklärung von Symptomen oder zur Vorsorge durchgeführt wird. Bei Personen mit Symptomen eines KRK wird neben der Koloskopie auch die CTK eingesetzt. Besonders bei unvollständiger optischer Koloskopie (OC) oder bei älteren und gebrechlichen PatientInnen, die sich keiner OC unterziehen können, oder bei Personen, die eine OC ablehnen, ist die CTK die diagnostische Methode der Wahl [1]. Neben der Diagnose und morphologischen Beurteilung von Dickdarmkarzinomen kann mittels CTK nahezu immer der gesamte prästenotische Dickdarm untersucht werden. Bei unvollständiger OC aufgrund stenosierender Tumoren ist das notwendig, um synchrone Zweitkarzinome oder synchrone Adenome des Kolons zu erkennen und die weitere Behandlung dementsprechend zu planen. Die gleichzeitige Erfassung der abdominalen Organe erlaubt eine Beurteilung der extrakolischen Tumorausbreitung.

Weitere KRK-assoziierte Einsatzmöglichkeiten für die CTK liegen in der opportunistischen Dickdarmkrebsvorsorge sowie auch in der Tumornachsorge, wenn die Koloskopie nicht vollständig durchführbar ist (Tab. 1).

**Tab. 1:** Einsatzmöglichkeiten der CT-Kolonographie beim kolorektalen Karzinom

**Diagnostische Fragestellungen**
– Abklärung von Symptomen → Optional zur Koloskopie
– Bei stenosierenden Karzinomen zur Tumorcharakterisierung und Untersuchung des prästenotischen Kolons → Ergänzend zur inkompletten Koloskopie
– Postoperativ zur Nachsorge bei kolorektalem Karzinom → Wenn Koloskopie inkomplett, kontraindiziert oder nicht möglich ist
**Screening**
– Keine Empfehlung als Erstlinientest in einem organisierten populationsbasierten Vorsorgeprogramm basierend auf FIT
– Opportunistische Dickdarmkrebsvorsorge, wo kein organisiertes FIT-basiertes Vorsorgeprogramm angeboten wird → Optional zur Koloskopie
– FIT-positive PatientInnen → Wenn Koloskopie inkomplett, kontraindiziert oder nicht möglich ist

*FIT* Fäkaler Immunochemischer Test

## Befundkriterien kolorektaler Karzinome

Die Bildanalyse einer CTK erfolgt routinemäßig mittels einer Kombination aus endoluminalen 3D-Ansichten und multiplanaren 2D-Schnittbildern. Suspekte kolorektale Befunde werden in der CTK grundsätzlich nach ihrem morphologischen Erscheinungsbild, der CT-Dichte und auch hinsichtlich ihrer Lagestabilität in den verschiedenen Scanpositionen beurteilt [11].

**Abb. 1:**
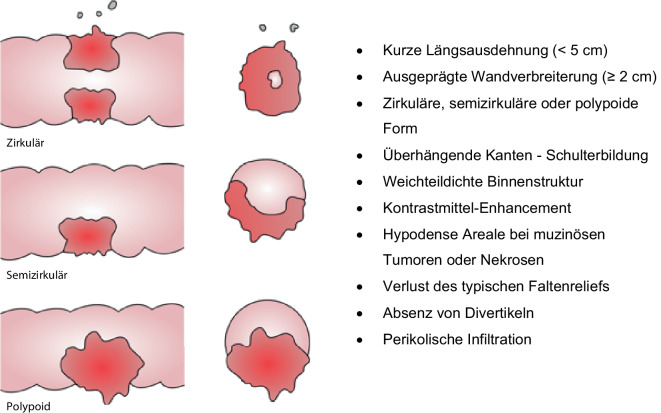
Wesentliche Befundkriterien der CT-Kolonographie (CTK) des kolorektalen Karzinoms (KRK)

Das zentrale radiologische Befundkriterium kolorektaler Karzinome ist die fokale tumoröse Wandverbreiterung des Dickdarms. Diese manifestiert sich typischerweise in Form einer zirkulären oder semizirkulären, oftmals stenosierenden Wandverbreiterung oder aber als fokale polypoide Raumforderung (Abb. 1).

**Abb. 2:**
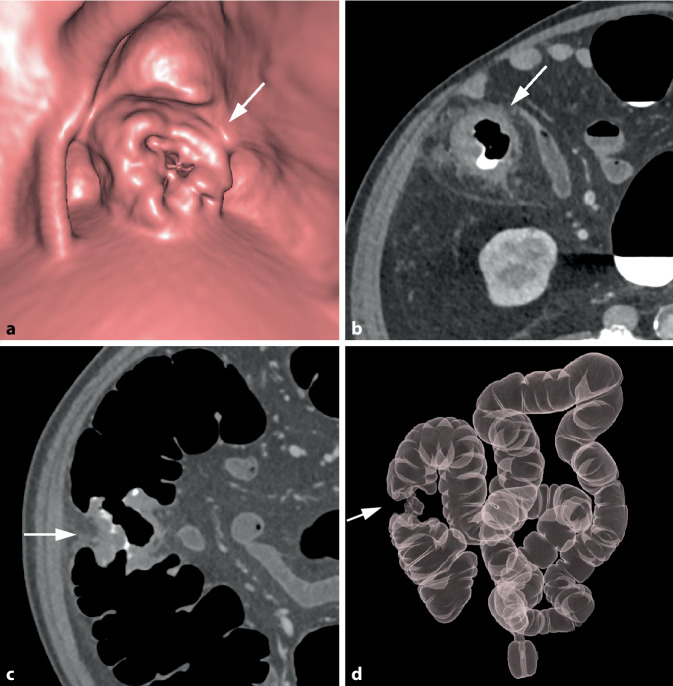
Zirkulär stenosierendes Karzinom im Colon ascendens bei inkompletter Koloskopie aufgrund einer Dickdarmelongation. **a** Die endoluminale 3D-Ansicht zeigt eine zirkulär stenosierende Raumforderung mit irregulärer Oberfläche und Schulterformation (*Pfeil*). **b** Das axiale 2D-Bild in Rückenlage zeigt die zirkuläre weichteildichte Wandverbreiterung (*Pfeil*). Es besteht eine unregelmäßige äußere Begrenzung mit nodulären Tumorinfiltrationen in das perikolische Fettgewebe sowie Fat Stranding als Zeichen der Wandüberschreitung (histologisch pT 3). **c** In der koronalen 2D-Ansicht ist die kurzstreckige weichteildichte tumoröse Wandverbreiterung mit Schulterformation (*Pfeil*) erkennbar. **d** In der globalen 3D-Ansicht zeigt sich die exakte Tumorlokalisation im Colon ascendens und der typische „Apple-core-Aspekt“ (*Pfeil*). Das Karzinom liegt bei 180 cm oberhalb des anorektalen Übergangs. Die Dickdarmgesamtlänge beträgt 220 cm

Bei zirkulär stenosierenden Tumoren umfasst die tumoröse Darmwandverbreiterung die gesamte Zirkumferenz des Darms (Abb. 2). Auf endoluminalen 3D-Ansichten erscheinen diese Karzinome meist als zirkuläre symmetrisch oder exzentrisch stenosierende Wandverbreiterungen. Das Ausmaß der Einengung des Darmlumens variiert und kann so ausgeprägt sein, dass diese Tumoren endoskopisch nicht passierbar sind.

**Abb. 3:**
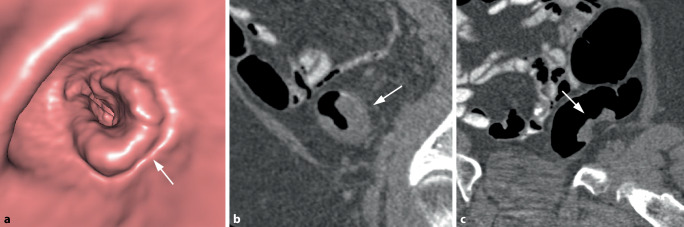
Semizirkuläres Karzinom im Colon sigmoideum. **a** Die endoluminale 3D-Ansicht und das korrespondierende parasagittale (**b**) und parakoronare 2D-Bild (**c**) zeigen die tumoröse Wandverbreiterung mit Schulterformation, die nur einen Teil der Dickdarmzirkumferenz betrifft (*Pfeil*). **c** Das parakoronare 2D-Bild zeigt die kurzstreckige Tumorlängsausdehnung und die homogene, weichteildichte Struktur (*Pfeil*)

**Abb. 4:**
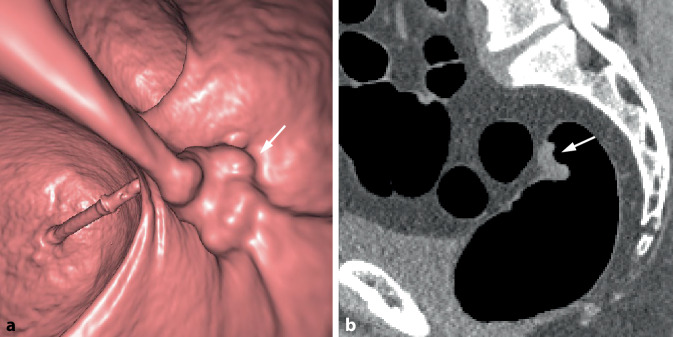
Sattelförmiges Karzinom im mittleren Rektum. **a** Die endoluminale 3D-Ansicht zeigt eine kleine sattelförmige Läsion im mittleren Rektum (*Pfeil*). **b** Das axiale 2D-Bild zeigt eine kurzstreckige semizirkuläre Wandverbreiterung mit Schulterbildung und weichteildichter Struktur (*Pfeil*)

Bei semizirkulären Tumoren nimmt die tumoröse Wandverdickung nur einen unterschiedlich großen Anteil der Zirkumferenz eines Darmsegments ein (Abb. 3). Bei kleineren Tumoren kann sich dabei in der 3D-Darstellung ein Aspekt ergeben, der an einen Pferdesattel erinnert (Abb. 4).

**Abb. 5:**
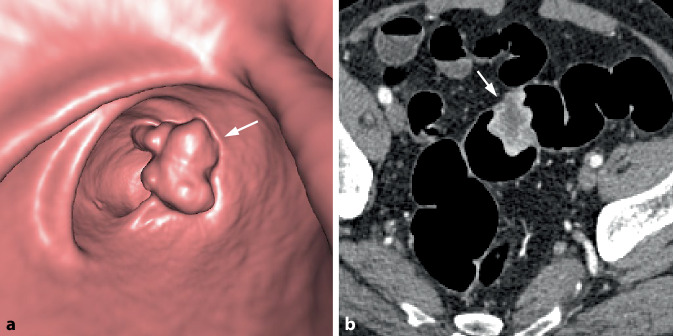
Polypoides Karzinom im Colon sigmoideum. **a** Die endoluminale 3D-Ansicht einer polypoiden Raumforderung mit lobuliertem Aspekt (*Pfeil*). **b** Das axiale 2D-Bild zeigt eine fokale polypoide Wandverbreiterung mit weichteildichter Struktur nach Kontrastmittelapplikation (*Pfeil*). Die zentrale Hypodensität spricht für eine muzinöse Komponente

Bei polypösen Tumoren finden sich meist große, breitbasige, sessile Raumforderungen von über 3 cm Größe, typischerweise mit einem lobulierten Aspekt (Abb. 5). Durch Tumornekrosen kann es zur Ausbildung von Ulzerationen kommen.

Die Oberfläche kolorektaler Karzinome ist meist unregelmäßig bzw. lobuliert, kann allerdings auch nodulär und selten glatt sein.

Die longitudinale Ausdehnung der tumorösen Wandverdickung ist bei Karzinomen typischerweise kurzstreckig (< 5 cm) [12]. Der Übergang zur normalen Darmwand ist meist abrupt mit Ausbildung von überhängenden Tumorrändern, die auch als Schulterbildung bezeichnet wird. Bei zirkulär stenosierenden Karzinomen ergibt sich so besonders auf 3D-Übersichtsbildern das vom Kolonkontrasteinlauf bekannte Bild einer „apple core lesion“ (Abb. 2d).

### 2D-Befundkriterien

**Abb. 6:**
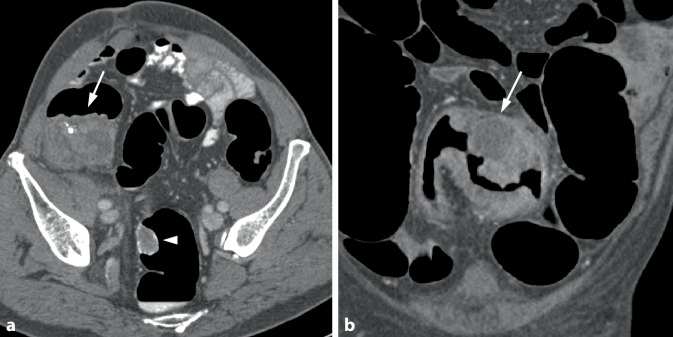
Muzinöse Tumoren und Tumornekrosen. **a** Parakoronares 2D-Bild eines muzinösen Zökumkarzinoms. Der polypoide Tumor weist eine inhomogene, teils hypodense Struktur auf (*Pfeil*). Zu beachten ist der große synchrone Polyp im distalen Sigma (*Pfeilspitze*). **b** Koronares 2D-Bild eines stenosierenden Sigmakarzinoms. Innerhalb der tumorösen Wandverbreiterung zeigt sich ein umschriebenes hypodenses Areal als Zeichen einer Tumornekrose bzw. Einschmelzung (*Pfeil*)

Auf 2D-CT-Bildern zeigen kolorektale Karzinome eine ausgeprägte Wandverbreiterung mit meist homogener weichteildichter Binnenstruktur. Sie überschreitet oftmals eine Breite von 2 cm. Die durchschnittlichen CT-Dichtewerte von Karzinomen liegen auf nativen 2D-Bildern bei rund 43 HU. Nach intravenöser Kontrastmittelapplikation zeigen kolorektale Karzinome ein deutliches Kontrastmittel-Enhancement auf ca. 91 HU [13]. Hypodense Areale innerhalb von Karzinomen können durch Tumornekrosen bzw. Einschmelzungen bedingt sein. Sie finden sich allerdings auch bei muzinösen Karzinomen (Abb. 6). Nach Fecal Tagging kann es an der Tumoroberfläche zu Ablagerungen von oralem Kontrastmittel, sog. „contrast coating“, kommen.

Die tumorösen Wandverbreiterungen führen zudem zu einer Architekturstörung der Darmwand mit Verlust der semilunaren Falten und Haustren [14]. Typischerweise fehlen auch Divertikel innerhalb der Tumoren, wenn diese in Segmenten mit Divertikelerkrankung lokalisiert sind [15].

### Lageabhängigkeit

Kolorektale Karzinome entspringen der Darmwand und sind im Gegensatz zu residualem Darminhalt lagestabile Raumforderungen, die in Bauch- und Rückenlage an derselben Stelle nachweisbar sein müssen. Dieses Kriterium ist auch für die Differenzialdiagnose von Pseudostenosen durch kollabierte Segmente und Spasmen essenziell. Die Bestätigung eines tumorsuspekten Befundes sollte in der CTK daher immer in beiden Scanpositionen erfolgen.

### Tumorstadium

Neben der Tumordetektion sollte in der CTK auch immer auf die lokale Ausbreitung des Tumors in Bezug auf perikolische Strukturen und Organe eingegangen werden.

Mit Ausnahme von T4-Tumoren hat das T‑Stadium beim Kolonkarzinom bisher allerdings wenig an der chirurgischen Behandlung geändert und war in der präoperativen Untersuchung von untergeordneter Bedeutung [16]. PatientInnen mit lokal fortgeschrittenen Tumoren können allerdings von einer neoadjuvanten Chemotherapie profitieren. Zu diesem Zweck werden zunehmend Hochrisikotumoren (Stadien T3c, T3d und T4) von Niedrigrisikotumoren (Stadien T1, T2, T3a und T3b) differenziert [17].

Die Beurteilung der lokalen Tumorausbreitung erfolgt im Weichteilfenster anstatt der sonst zur Auswertung gebräuchlichen weiten Kolon-Fenstereinstellungen. Dabei wird auf die äußere Begrenzung des betroffenen Darmsegments zum perikolischen Fettgewebe geachtet. Die Tiefe der Tumorinvasion wird am besten am Tumorquerschnitt orthogonal zur Längsachse des betroffenen Darmsegments dargestellt.

Bei Tumoren, die auf die Darmwand limitiert sind (T1 und T2), weist die äußere Darmwand einen regelmäßigen Verlauf mit einer scharfen äußeren Begrenzung zum Fettgewebe auf. Das perikolische Fettgewebe ist unauffällig, Fetttrennungslinien sind erhalten.

Für die Ausbreitung des Tumors über die Darmwand hinaus (T3) sprechen neben unspezifischen, perikolischen Fettgewebsverdichtungen besonders eine unscharfe oder unregelmäßig verlaufende äußere Begrenzung des Darmsegments mit nodulärer Tumorinfiltration in das angrenzende Fettgewebe (Abb. 2b). Die Beurteilung der Tiefenausdehnung der perikolischen Tumorinfiltration ermöglicht eine Subklassifikation von T3-Tumoren in T3a- (< 1 mm), T3b- (< 5 mm), T3c- (5–15 mm) und T3d-Tumoren (> 15 mm).

Eine Tumorperforation des viszeralen Peritoneums führt zur peritonealen Karzinomatose (T4a). Die direkte Infiltration in andere Organe (T4b) ist durch Aufhebung der Fetttrennungslinien zwischen Tumor und angrenzenden Organen charakterisiert.

### Segmentale Tumorlokalisation

Die CTK liefert eine präzise Information über die segmentale Lokalisation von Tumoren im Kolon, die unverfälschte „In-vivo-Dickdarmgesamtlänge“, und die anatomisch-topografischen Gegebenheiten des Kolons (Abb. 2d). Derartige Informationen können für die Planung des chirurgischen Vorgehens hilfreich sein. Bei der Koloskopie wird hingegen der Dickdarm oftmals über das Endoskop geschoben, wodurch Längenangaben vom CTK-Befund deutlich abweichen können [18].

## Evaluation des prästenotischen Kolons zur Diagnose synchroner Neoplasien

Bei PatientInnen, bei denen ein kolorektales Karzinom diagnostiziert wurde, besteht ein hohes Risiko für synchrone Dickdarmneoplasien. Synchrone Zweitkarzinome finden sich in bis zu 6 % und synchrone adenomatöse Polypen in bis zu 39 % [19, 20]. Nichterkannte synchrone Neoplasien können die Prognose verschlechtern und zusätzliche operative Eingriffe notwendig machen bzw. eine kurative Therapie verhindern.

Stenosierende Karzinome können mit Endoskopen nicht immer passiert werden, sodass Teile des Kolons vor der Behandlung koloskopisch nicht untersuchbar sind. Die CTK ist in diesen Fällen eine zuverlässige und nichtinvasive Option, die nahezu immer eine vollständige Untersuchung des gesamten Dickdarms erlaubt. Die Sensitivität und der negative Vorhersagewert der CTK für synchrone prästenotische Karzinome und Adenome ist in zahlreichen Studien sehr hoch [20–22]. Die Untersuchung der prästenotischen Dickdarmabschnitte ist folglich neben der Beurteilung des Tumors eine wesentliche Aufgabe der CTK [1].

Nach frustraner Koloskopie ist die CTK am selben Tag im Zuge der gleichen Vorbereitung anzustreben, sofern keine Polypektomie oder eine mukosale Resektion stattgefunden haben. Essenziell ist allerdings die Gabe von iodhaltigem Kontrastmittel (beispielsweise 50 ml Gastrografin®) zum Fecal Tagging mindestens 3 h vor der CTK. Zur Beurteilung extrakolischer Tumormanifestationen wird weiters beim CT-Scan in Rückenlage die intravenöse Kontrastmittelapplikation und ggf. die Erweiterung der Untersuchung auf den Thorax empfohlen.

## CTK zur Dickdarmkrebsvorsorge

Mit der hohen Sensitivität für fortgeschrittene Dickdarmneoplasien und der minimalen Invasivität erfüllt die CT-Kolonographie wesentliche Kriterien einer Vorsorgeuntersuchung. Allerdings muss der Nachweis erbracht werden, dass sich durch ihren Einsatz auch die Inzidenz und Mortalität der Erkrankung in einer Population reduzieren lassen. Prospektive, randomisierte Langzeitstudien mit diesen Endpunkten wurden für die CTK bisher nicht veröffentlicht. Derartige Effekte wurden allerdings bisher direkt nur bei der flexiblen Sigmoidoskopie und bei Blutstuhltests nachgewiesen [23].

Die Gestaltung von KRK-Screenings ist weltweit verschieden. In den USA empfehlen die United States Preventive Service Task Force (USPSTF) und die American Cancer Society die CTK neben anderen Testoptionen zur opportunistischen Dickdarmkrebsvorsorge. Diese ist individuell und personenbezogen. Ab 2025 werden die Kosten für die Vorsorge-CT-Kolonographie auch durch das staatliche Krankenversicherungsprogramm Medicare übernommen [24].

In Europa wurden gemäß Empfehlung der Europäischen Kommission in zahlreichen Ländern organisierte, populationsbasierte Programme zur Dickdarmkrebsvorsorge eingeführt [25]. Dabei werden asymptomatische Personen im Alter von 45 bis 75 Jahren eingeladen, sich einer Vorsorgeuntersuchung zu unterziehen. Die meisten dieser Vorsorgeprogramme basieren primär auf Blut-Stuhltests, in erster Linie auf dem fäkalen immunochemischen Test (FIT), bei positivem Testergebnis gefolgt von einer Koloskopie. Dieser Test erweist sich in einem populationsbasierten Einsatz aufgrund der hohen Akzeptanz und Teilnahme bei wiederholter Durchführung als effektiv [26].

Die CTK wird folglich nicht als Erstlinientest im Rahmen organisierter populationsbasierter Programme empfohlen. Eine starke Empfehlung gibt es allerdings zur Untersuchung von Personen mit positivem Stuhltest, bei denen die OC nicht vollständig durchführbar ist, oder die sich keiner OC unterziehen können oder möchten [1]. FIT-positive Personen haben ein deutlich höheres Risiko für fortgeschrittene Dickdarmneoplasien und benötigen eine weiterführende Abklärung des Befundes [27, 28]. Diese Vorgangsweise wird bereits in Vorsorgeprogrammen in England und Irland verfolgt und wurde in den Niederlanden nachträglich implementiert [29, 30].

Wo keine populationsbasierten FIT-Programme etabliert sind, besteht seitens der ESGAR und ESGE auch eine Empfehlung zur opportunistischen KRK-Vorsorge. Allerdings werden die Kosten für diese Indikation in Europa von den Krankenversicherungen meistens nicht übernommen.

### Morphologische Besonderheiten von mittels Vorsorge-CTK diagnostizierten Frühkarzinomen

Mittels Screening werden Krebserkrankungen typischerweise in einem früheren asymptomatischen Stadium erkannt als Tumoren, die erst aufgrund von Symptomen festgestellt werden. Konsekutiv unterscheiden sie sich auch radiomorphologisch von symptomatischen Tumoren [31].

**Abb. 7:**
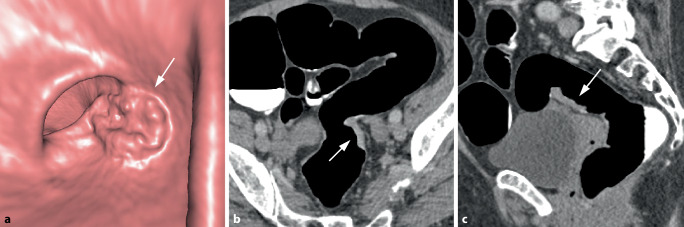
Flaches Karzinom im oberen Rektum (G2, pT2, L0, V0, R0, pN0). **a** Die endoluminale 3D-Ansicht zeigt eine flache Wanderhabenheit mit nodulärer Oberfläche (*Pfeil*). **b** Das axiale und sagittale 2D-Bild (**c**) zeigen eine plaqueförmige Wandverbreiterung mit weichteildichter Struktur (*Pfeil*)

Asymptomatische Tumoren sind wesentlich kleiner und von geringerem Volumen als symptomatische KRK und daher radiologisch deutlich weniger auffällig und schwerer erkennbar. Der morphologische Aspekt ist eher polypös oder sattelförmig und seltener zirkulär oder stenosierend. Mittels Screening erkannte KRK weisen auch ein früheres radiologisches Tumorstadium auf. Suspekte Lymphknoten oder vaskuläre Invasionen sind seltener als bei symptomatischen Karzinomen (Abb. 7).

Symptomatische Tumoren sind hingegen größer und bereits invasiver. Sie weisen häufiger eine zirkuläre Morphologie auf und engen das Darmlumen in größerem Ausmaß ein (Abb. 2; [32]).

## CTK zur Nachsorge nach Resektion eines kolorektalen Karzinoms

Bei PatientInnen mit reseziertem kolorektalen Karzinom besteht ein 30 %iges Risiko eines Rezidivs, weshalb Nachsorgeuntersuchungen erforderlich sind [33, 34]. Eine frühe Erkennung und Behandlung von Tumorrezidiven kann die Überlebensrate erhöhen.

Rezidive können entweder im Kolon oder extrakolisch lokalisiert sein. Die CTK ermöglicht bei der Mehrzahl der PatientInnen eine vollständige endoluminale Beurteilung des gesamten restlichen Kolons und erlaubt neben der Darstellung des postoperativen Situs die Diagnose von lokoregionären Rezidiven und metachronen Kolonneoplasien. Gleichzeitig ermöglicht die i.v.-kontrastmittelverstärkte CTK auch die Beurteilung der abdominalen Organe hinsichtlich Fernmetastasen, wodurch Nachsorgeprozesse vereinfacht werden könnten.

Da sich in Studien anfangs eine gute technische Durchführbarkeit und sehr hohe Sensitivität und Spezifität für Anastomosenrezidive und metachrone Karzinome sowie eine moderate Sensitivität für Polypen [35] zeigte, waren die Ergebnisse in einer späteren prospektiven, multizentrischen Studie mit einer Sensitivität von nur 44 % für Polypen ≥ 6 mm und 76,9 % für Polypen ≥ 10 mm nicht zufriedenstellend [36]. Als Ursache wurde die schlechtere Distendierbarkeit des postoperativen Kolons vermutet. Überdies wurde die CTK von den KarzinompatientInnen mehrheitlich nicht bevorzugt [37].

Aufgrund der unzureichenden Datenlage empfehlen ESGAR und ESGE keinen primären Einsatz der CTK für diese Indikation. Sie kann allerdings bei den Patienten zur KRK-Nachsorge eingesetzt werden, bei denen die optische Koloskopie kontraindiziert oder nicht durchführbar ist [1].

### Fehlermöglichkeiten und Differenzialdiagnosen

#### Falsch-negative Befunde

Werden kolorektale Karzinome in einem definierten Zeitraum (meist 3 Jahre) nach einer „unauffällig“ befundeten bildgebenden Untersuchung entdeckt, kann davon ausgegangen werden, dass sie, oder eine Vorstufe, zum Zeitpunkt dieser Untersuchung bereits vorgelegen sind. Solche Tumoren werden „post-imaging colorectal cancers“ (PICRC) oder Intervallkarzinome genannt. Sie sind ein Qualitätsindikator für einen diagnostischen Test.

**Abb. 8:**
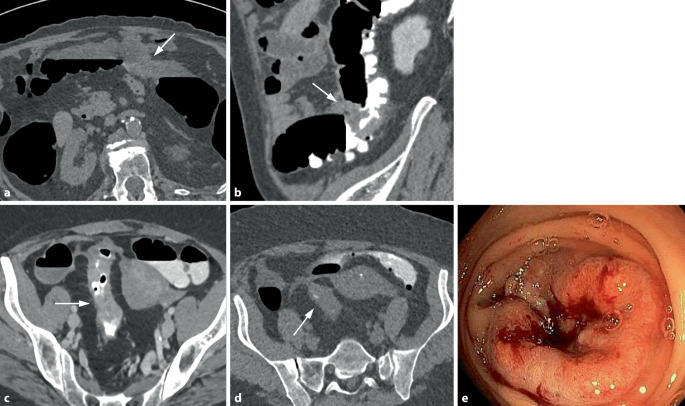
Fehldiagnosen eines kolorektalen Karzinoms (KRK) aufgrund technischer Fehler. **a** Fehlendes Fecal Tagging: Das zirkulär stenosierende Karzinom im Colon transversum ist innerhalb der nicht kontrastmittelmarkierten Flüssigkeit kaum erkennbar (*Pfeil*). **b** Anderer Patient mit Fecal Tagging: Das zirkulär stenosierende Karzinom im Colon descendens ist innerhalb der kontrastmittelmarkierten Flüssigkeit gut erkennbar (*Pfeil*). **c**, **d** Unzureichende Sigmadistension in beiden Scanpositionen. Das stenosierende Karzinom im kollabierten Darmsegment ist leicht zu übersehen (*Pfeil*). **e** Koloskopisches Korrelat

In einer Metaanalyse einer englischen Arbeitsgruppe hat sich gezeigt, dass die Rate an PICRC der CTK sehr gering und mit 4,4 % vergleichbar mit der der Koloskopie (2,9–8,6 %) ist [9]. Die retrospektive Aufarbeitung der übersehenen Karzinome in dieser Studie hat ergeben, dass 61 % retrospektiv sichtbar und somit Perzeptionsfehler ursächlich waren. Für die übrigen Fehlbefunde waren Fehler in der Untersuchungstechnik und beim PatientInnenmanagement verantwortlich (Abb. 8). Retrospektiv gar nicht abgrenzbare „okkulte“ Karzinome waren hingegen sehr selten. Fehldiagnosen können durch Optimierung der Untersuchungstechnik mittels spezifischem Training der Untersucher vermieden werden [38, 39].

#### Entzündliche Stenosen

Entzündliche Stenosen, wie beispielsweise bei der Divertikelerkrankung, weisen überlappende Befundkriterien auf und können eine differenzialdiagnostische Herausforderung sein. Sie zeigen im Gegensatz zu stenosierenden Karzinomen meist eine geringer ausgeprägte stenosierende Darmwandverdickung, die typischerweise langstreckig (meist > 10 cm) ist. Der Übergang der Wandverbreiterung in das Niveau der normalen Darmwand verläuft kontinuierlich und typischerweise ohne Schulterbildung [12]. Differenzialdiagnostisch sprechen zudem die Präsenz von Divertikeln sowie erhaltene Darmfalten innerhalb der Wandverbreiterung für die entzündliche Genese.

**Abb. 9:**
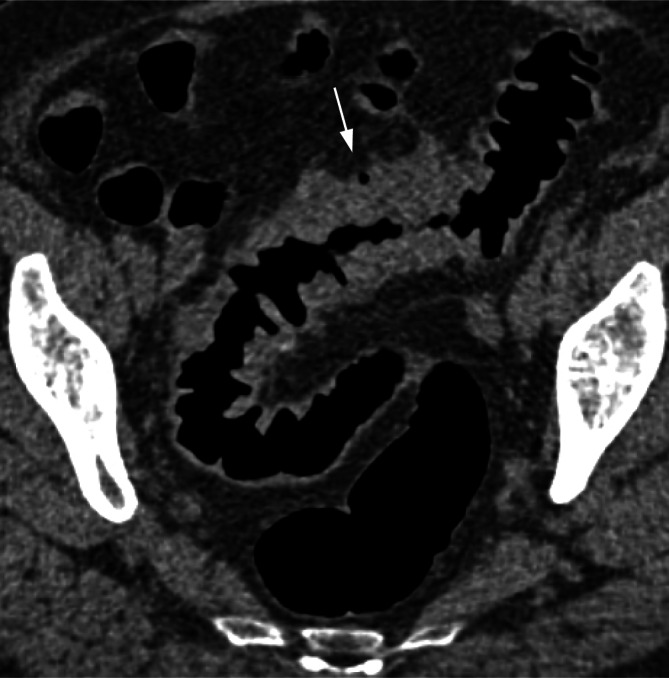
Chronische Divertikelerkrankung mit postentzündlicher Sigmastenose. Das koronale 2D-Bild zeigt eine zirkuläre langstreckige Wandverbreiterung ohne Schulterbildung. Erkennbar ist die teils erhaltene Faltenstruktur sowie ein Divertikel (*Pfeil*)

Begleitend finden sich entzündliche Veränderungen des perikolischen Fettgewebes und des Mesenteriums (Abb. 9) [14, 15].

#### Spasmen

Spasmen sind zirkuläre Kontraktionen der Darmwand, die in der CTK stenotische tumorartige Läsionen simulieren können [40]. Anders als bei invasiven Tumoren findet sich bei Spasmen eine glatte Begrenzung des betroffenen Wandsegments zum umliegenden Fettgewebe, das überdies regulär strukturiert ist. Spasmen lösen sich oft während der Untersuchung und sind nach Umlagerung der PatientInnen in die zweite Scanposition meist nicht mehr nachweisbar.

## Klinische Nutzung der CT-Kolonographie

Obwohl die CTK in interdisziplinäre Richtlinien bzw. Empfehlungen aufgenommen wurde, wird diese Methode vielerorts vergleichsweise selten angewendet [24]. Dabei überwiegen diagnostische Indikationen gegenüber der Vorsorge, deren Kosten von den Krankenkassen meist nicht übernommen werden. Trotz der vorliegenden Evidenz zur diagnostischen Genauigkeit und Sicherheit der CTK konnte sich die Untersuchung interdisziplinär noch nicht überall etablieren. Indikationen, Leistungsfähigkeit und auch die Verfügbarkeit der Methode sind zuweisenden ÄrztInnen und PatientInnen nicht immer ausreichend bekannt. Hinzu kommt nicht nur die zögerliche Akzeptanz der CTK durch EndoskopikerInnen, sondern auch die vielerorts gute Verfügbarkeit der Koloskopie. Die Untersuchung ist zudem zeitaufwändig und erfordert ein spezifisches Training zur Durchführung und Befundung. Sie wird von radiologischen Einrichtungen nicht immer angeboten [24, 41].

Dass die CTK in der PatientInnenversorgung wertvolle Dienste leisten kann, zeigt sich in anderen Ländern, wie beispielsweise im Vereinigten Königreich. Dort werden jährlich über 120.000 CTK durchgeführt. An manchen Standorten sind das bis zu 3000 Untersuchungen [42]. Innerhalb des National Bowel Cancer Screening Programms waren 6,1 % der nach einem positiven Stuhltest durchgeführten Folgeuntersuchungen CTK [38]. Analog zur Koloskopie wurden für die CTK Standards zur spezifischen Ausbildung und Instrumente zur Qualitätskontrolle geschaffen [43].

## Schlusswort

Die CTK ist die beste minimal-invasive Untersuchung zur Abklärung kolorektaler Neoplasien. Sie wird für diese Indikation als radiologische Untersuchung der Wahl empfohlen [1]. Aufgrund ihrer hohen Sensitivität für kolorektale Karzinome und fortgeschrittene Adenome eignet sie sich besonders für PatientInnen mit KRK-Symptomen oder Tumorverdacht. Sie kann aber auch zur opportunistischen Dickdarmkrebsvorsorge eingesetzt werden. Bei KarzinompatientInnen wird die CTK zur Beurteilung des Lokalbefundes und zur Erkennung synchroner Neoplasien in prästenotischen Dickdarmsegmenten eingesetzt. Sie erlaubt auch die Beurteilung extrakolischer Tumormanifestationen. Trotz der vorliegenden Evidenz zur diagnostischen Genauigkeit und Sicherheit wird die CTK vergleichsweise selten eingesetzt.

## Fazit für die Praxis

- Die diagnostische Performance der Computertomographie-Kolonographie (CTK) für kolorektale Karzinome (KRK) und fortgeschrittene Adenome ist mit jener der Koloskopie vergleichbar.
- Fehldiagnosen von Karzinomen sind bei der CTK sehr selten; diese sind entweder durch Perzeptionsfehler oder technische Fehler bedingt.
- Die CTK stellt eine wichtige Ergänzung zur Koloskopie dar und eignet sich besonders für PatientInnen mit Tumorverdacht oder erhöhtem Neoplasierisiko, wenn eine Koloskopie nicht oder nicht vollständig durchgeführt werden kann.
- Die CTK eignet sich auch zur individuellen opportunistischen Dickdarmkrebsvorsorge. In FIT-basierten Vorsorgeprogrammen (fäkaler immunochemischer Test) sollte sie positiv getesteten Personen angeboten werden, bei denen eine Koloskopie nicht erfolgreich durchführbar ist.
- Trotz der vorliegenden Evidenz zur diagnostischen Genauigkeit und Sicherheit konnte sich die CTK interdisziplinär noch nicht ausreichend gut etablieren.

## References

[1] Spada C, Hassan C, Bellini D et al (2021) Imaging alternatives to colonoscopy: CT colonography and colon capsule. European Society of Gastrointestinal Endoscopy (ESGE) and European Society of Gastrointestinal and Abdominal Radiology (ESGAR) Guideline – Update 2020. Eur Radiol 31:2967–2982 33104846 10.1007/s00330-020-07413-4

[2] Neri E, Halligan S, Hellstrom M et al (2013) The second ESGAR consensus statement on CT colonography. Eur Radiol 23:720–729 22983280 10.1007/s00330-012-2632-x PMC3563960

[3] American College of Radiology (2019) ACR-SAR-SCBT-MR Practice Parameter for the Performance of CT Colonography in Adults. In: ACR practice guideline 2014. American College of Radiology,

[4] Yee J, Dachman A, Kim DH et al (2024) CT Colonography Reporting and Data System (C-RADS): Version 2023 Update. Radiology 310:e232007 38289209 10.1148/radiol.232007

[5] Mang T, Lampichler K, Scharitzer M (2023) CT colonography : Technique and indications. Radiol 63:418–428 10.1007/s00117-023-01153-4 PMC10234944 37249607

[6] Pickhardt PJ, Hassan C, Halligan S et al (2011) Colorectal cancer: CT colonography and colonoscopy for detection—systematic review and meta-analysis. Radiology 259:393–405 21415247 10.1148/radiol.11101887 PMC3079122

[7] Atkin W, Dadswell E, Wooldrage K et al (2013) Computed tomographic colonography versus colonoscopy for investigation of patients with symptoms suggestive of colorectal cancer (SIGGAR): a multicentre randomised trial. Lancet 381:1194–1202 23414650 10.1016/S0140-6736(12)62186-2

[8] Tutein Nolthenius CJ, Boellaard TN, De Haan MC et al (2016) Computer tomography colonography participation and yield in patients under surveillance for 6–9 mm polyps in a population-based screening trial. Eur Radiol 26:2762–2770 26560732 10.1007/s00330-015-4081-9 PMC4927597

[9] Obaro AE, Plumb AA, Fanshawe TR et al (2018) Post-imaging colorectal cancer or interval cancer rates after CT colonography: a systematic review and meta-analysis. Lancet Gastroenterol Hepatol 3:326–336 29472116 10.1016/S2468-1253(18)30032-3

[10] International Agency for Research on Cancer (2022) Globocan 2022: Cancer Fact Sheets—Colorectal Cancer. https://gco.iarc.who.int/en. Zugegriffen: 16.02.2025

[11] Mang T, Gryspeerdt S, Schima W et al (2013) Evaluation of colonic lesions and pitfalls in CT colonography: a systematic approach based on morphology, attenuation and mobility. Eur J Radiol 82:1177–1186 22817848 10.1016/j.ejrad.2012.05.024

[12] Chintapalli KN, Chopra S, Ghiatas AA et al (1999) Diverticulitis versus colon cancer: differentiation with helical CT findings. Radiology 210:429–435 10207426 10.1148/radiology.210.2.r99fe48429

[13] Oto A, Gelebek V, Oguz BS et al (2003) CT attenuation of colorectal polypoid lesions: evaluation of contrast enhancement in CT colonography. Eur Radiol 13:1657–1663 12835982 10.1007/s00330-002-1770-y

[14] Lips LM, Cremers PT, Pickhardt PJ et al (2015) Sigmoid cancer versus chronic diverticular disease: differentiating features at CT colonography. Radiology 275:127–135 25426771 10.1148/radiol.14132829

[15] Gryspeerdt S, Lefere P (2012) Chronic diverticulitis vs. colorectal cancer: findings on CT colonography. Abdom Imaging 37:1101–1109 22366853 10.1007/s00261-012-9858-6

[16] Tamandl D, Mang T, Ba-Ssalamah A (2018) Imaging of colorectal cancer – the clue to individualized treatment. Innov Surg Sci 3:3–15 31579761 10.1515/iss-2017-0049 PMC6754048

[17] Horvat N, Raj A, Liu S et al (2019) CT Colonography in Preoperative Staging of Colon Cancer: Evaluation of FOxTROT Inclusion Criteria for Neoadjuvant Therapy. AJR Am J Roentgenol 212:94–102 30422707 10.2214/AJR.18.19928 PMC7959265

[18] Duncan JE, Mcnally MP, Sweeney WB et al (2009) CT colonography predictably overestimates colonic length and distance to polyps compared with optical colonoscopy. AJR Am J Roentgenol 193:1291–1295 19843744 10.2214/AJR.09.2365

[19] Flor N, Zanchetta E, Di Leo G et al (2018) Synchronous colorectal cancer using CT colonography vs. other means: a systematic review and meta-analysis. Abdom Radiol 10.1007/s00261-018-1658-1 29948053

[20] Flor N, Ceretti AP, Luigiano C et al (2020) Performance of CT Colonography in Diagnosis of Synchronous Colonic Lesions in Patients With Occlusive Colorectal Cancer. AJR Am J Roentgenol 214:348–354 31670584 10.2214/AJR.19.21810

[21] Neri E, Giusti P, Battolla L et al (2002) Colorectal cancer: role of CT colonography in preoperative evaluation after incomplete colonoscopy. Radiology 223:615–619 12034925 10.1148/radiol.2233010928

[22] Park SH, Lee JH, Lee SS et al (2012) CT colonography for detection and characterisation of synchronous proximal colonic lesions in patients with stenosing colorectal cancer. Gut 61:1716–1722 22115824 10.1136/gutjnl-2011-301135

[23] Lin JS, Perdue LA, Henrikson NB et al (2021) Screening for Colorectal Cancer: Updated Evidence Report and Systematic Review for the US Preventive Services Task Force. JAMA 325:1978–1998 34003220 10.1001/jama.2021.4417 PMC13509038

[24] Kim DH, Obaro AE, Taylor SA et al (2025) CT Colonography for Colorectal Cancer Prevention and Detection: Integration Into Clinical Practice, From the AJR Special Series on Screening. AJR Am J Roentgenol 10.2214/AJR.25.32633 40071901

[25] Segnan N, Patnick J, Karsa L (2010) European guidelines for quality assurance in colorectal cancer screening and diagnosis. Publications Office of the European Union, Luxenbourg 10.1055/s-0032-1309822 23012113

[26] Sali L, Ventura L, Mascalchi M et al (2022) Single CT Colonography versus three Rounds of Faecal Immunochemical Test for Population-based Screening of Colorectal Cancer: The SAVE Randomised Clinical Trial. Lancet Gastroenterol Hepatol 10.1016/S2468-1253(22)00269-2 36116454

[27] Van Rossum LG, Van Rijn AF, Laheij RJ et al (2008) Random comparison of guaiac and immunochemical fecal occult blood tests for colorectal cancer in a screening population. Gastroenterology 135:82–90 18482589 10.1053/j.gastro.2008.03.040

[28] Zorzi M, Fedato C, Naldoni C et al (2009) Screening for colorectal cancer in Italy: 2007 survey. Epidemiol Prev 33:57–74 19776487

[29] Plumb AA, Halligan S, Nickerson C et al (2013) Use of CT colonography in the English Bowel Cancer Screening Programme. Gut 63(6):964. 10.1136/gutjnl-2013-304697 23955527 10.1136/gutjnl-2013-304697 PMC4033278

[30] Lammertink MHA, Huisman JF, Bernsen MLE et al (2021) Implications of colonic and extra-colonic findings on CT colonography in FIT positive patients in the Dutch bowel cancer screening program. Scand J Gastroenterol 56:1337–1342 34506230 10.1080/00365521.2021.1966091

[31] Plumb AA, Pathiraja F, Nickerson C et al (2016) Appearances of screen-detected versus symptomatic colorectal cancers at CT colonography. Eur Radiol 26:4313–4322 27048534 10.1007/s00330-016-4293-7 PMC5101282

[32] Lee MH, Hinshaw JL, Kim DH et al (2016) Symptomatic Versus Asymptomatic Colorectal Cancer: Predictive Features at CT Colonography. Acad Radiol 23:712–717 26852246 10.1016/j.acra.2015.12.009

[33] Manfredi S, Bouvier AM, Lepage C et al (2006) Incidence and patterns of recurrence after resection for cure of colonic cancer in a well defined population. Br J Surg 93:1115–1122 16804870 10.1002/bjs.5349

[34] Schoemaker D, Black R, Giles L et al (1998) Yearly colonoscopy, liver CT, and chest radiography do not influence 5‑year survival of colorectal cancer patients. Gastroenterology 114:7–14 9428212 10.1016/s0016-5085(98)70626-2

[35] Kim HJ, Park SH, Pickhardt PJ et al (2010) CT Colonography for Combined Colonic and Extracolonic Surveillance after Curative Resection of Colorectal Cancer. Radiology 257:697–704 20876390 10.1148/radiol.10100385

[36] Weinberg DS, Pickhardt PJ, Bruining DH et al (2018) Computed Tomography Colonography vs Colonoscopy for Colorectal Cancer Surveillance After Surgery. Gastroenterology 154(e924):927–934 29174927 10.1053/j.gastro.2017.11.025 PMC5847443

[37] Weinberg DS, Mitnick J, Keenan E et al (2019) Post-operative colorectal cancer surveillance: preference for optical colonoscopy over computerized tomographic colonography. Cancer Causes Control 30:1269–1273 31531798 10.1007/s10552-019-01231-w PMC7534185

[38] NHS England (2024) Bowel Cancer Screening Annual Report 2021 to 2022. https://www.gov.uk/government/publications/bowel-cancer-screening-annual-report-2021-to-2022/bowel-cancer-screening-annual-report-2021-to-2022. Zugegriffen: 22.02.2025

[39] Obaro AE, Plumb AA, Halligan S et al (2022) Colorectal Cancer: Performance and Evaluation for CT Colonography Screening—A Multicenter Cluster-randomized Controlled Trial. Radiology 303:361–370 35166585 10.1148/radiol.211456

[40] Mang T, Maier A, Plank C et al (2007) Pitfalls in multi-detector row CT colonography: a systematic approach. Radiographics 27:431–454 17374862 10.1148/rg.272065081

[41] Kim DH (2018) CT colonography: the ideal colorectal cancer screening test. Abdom Radiol 43:515–516 10.1007/s00261-018-1514-3 29460039

[42] Duxbury O, Burling D, Muckian J et al (2021) Meeting the new joint British Society of Gastrointestinal and Abdominal Radiology and Royal College of Radiologists CT colonography standards: a 6-year experience. Clin Radiol 76:665–673 34148642 10.1016/j.crad.2021.05.007

[43] British Society of Gastrointestinal and Abdominal Radiology and The Royal College of Radiologists (2021) Standards of practice for computed tomographycolonography( CTC). Joint Guidance from the British Society of Gastrointestinal and Abdominal Radiology and the Royal College of Radiologists. https://www.rcr.ac.uk/our-services/all-our-publications/clinical-radiology-publications/standards-of-practice-for-computed-tomography-colonography-ctc. Zugegriffen: 25.02.2025

